# High Prevalence of Anal Sexually Transmitted Infections among Men Who Have Sex with Men and Transgender Women Attending a Clinic for Prevention of Anal Cancer in Salvador, Brazil

**DOI:** 10.3390/pathogens12111297

**Published:** 2023-10-30

**Authors:** Ivana Luz, Eda Vinhaes, Isabela Cruz, Ana Gabriela Travassos, Estela Luz, Eduardo Martins Netto, Carlos Brites

**Affiliations:** 1 Fundação Bahiana de Infectologia, Salvador 40110-060, Brazil; ivana.luz@ig.com.br (I.L.); eda.vinhaes@yahoo.com.br (E.V.); eluz5@yahoo.com.br (E.L.); nettoeduardom@hotmail.com (E.M.N.); 2 Programa de Pós-Graduação em Medicina e Saúde, Universidade Federal da Bahia, Salvador 40110-060, Brazil; 3 Hospital Universitário Professor Edgard Santos, UFBA-EBSERH, Salvador 40110-060, Brazil; idmcruz@gmail.com; 4 School of Medicine, Universidade Estadual da Bahia, Salvador 40110-060, Brazil; agtravassos@yahoo.com.br; 5 School of Medicine, Universidade Federal da Bahia, Salvador 40110-060, Brazil

**Keywords:** HIV, MSM, Sexually Transmitted Infections

## Abstract

Men who have sex with men (MSM) and transgender women (TGW) are highly vulnerable to anal sexually transmitted infections (STIs). **Objectives**—to evaluate the prevalence of anal STIs among MSM and TGW attending a referral clinic for anal cancer prevention. **Methods**—MSM and TGW attending a medical visit for high-resolution anoscopy in Salvador, Brazil, from February 2021 to June 2022 were screened for HPV, gonorrhea, and chlamydial infection by PCR of anal swab and by serum VDRL titration for syphilis screening. They also responded to a questionnaire on sociodemographic characteristics and sexual behavior. **Results**—we evaluated 141 participants: 117 (82.9%) MSM, 9 (6.4%) bisexual men (BSM), and 15 (10.6%) TGW. Most (111/141, 78.7%) were older than 30 years, 89 (63.1%) had over 12 years of education, and 124 (87.9%) had a family income of up to five minimum wages. At least one STI was detected in 112 (79.4%) of the participants (86.7% among TGW). HIV infection was detected in 102 (72.3%) participants; HIV frequency was higher in BSM (7/9, 88.9%) and in MSM (89/116, 76.1%) than in TGW (5/15, 33.3%). A lower income (*p* = 0.004) was predictive of anal STIs, while syphilis was significantly more frequent among participants with HIV (29.1% vs. 5,3%, for HIV positive and negative, respectively, *p* = 0.002). Presenting at least one active STI was also associated with having had group sex in the last year (*p* = 0.03) and with use of sexualized drugs (*p* = 0.02). **Conclusions**—MSM and TGW present a high vulnerability to anal STIs. Number of sexual partners, use of sexualized drugs, and lower income are predictive of a higher risk of acquiring an STI in such populations.

## 1. Introduction

Sexually transmitted infections (STIs) are a significant public health concern worldwide, affecting individuals across diverse populations and communities. Among specific populations, men who have sex with men (MSM) and transgender women (TGW) face unique challenges in terms of the prevalence and risk factors associated with anal STIs [1,2]. Anal intercourse is a common sexual practice within these communities, making them particularly vulnerable to the transmission of STIs through this route [3,4]. Understanding the prevalence and risk factors associated with anal STIs in MSM and TGW is crucial for effective prevention, diagnosis, and treatment strategies.

Men who have sex with men represent a population that experiences a higher burden of anal STIs compared to the general population. The prevalence of STIs such as gonorrhea, chlamydia, syphilis, and human papillomavirus (HPV) is significantly elevated among MSM who engage in receptive anal intercourse [3,4]. This increased risk can be attributed to several factors, including the biological vulnerability of the rectal mucosa, which can be easily traumatized during anal sex, facilitating the transmission of STIs. Moreover, a higher number of sexual partners, inconsistent condom use, and overlapping sexual networks contribute to the higher prevalence of anal STIs in this population [5].

Another population that also face a disproportionate burden of anal STIs is transgender women, particularly those who engage in receptive anal intercourse. Like MSM, transgender women are at increased risk due to the biological vulnerability of the rectal mucosa and the presence of multiple risk factors. Transgender women often face unique challenges related to healthcare access, discrimination, and stigma, which can further exacerbate their vulnerability to STIs [6]. Limited availability of culturally competent healthcare services and inadequate knowledge about sexual health contribute to barriers in the prevention, testing, and treatment of anal STIs in this population.

The risk factors for anal STIs in MSM and transgender women extend beyond individual-level behaviors. Social and structural determinants play a significant role in shaping the risk environment for these populations. Homophobia, transphobia, and stigma surrounding same-sex relationships and transgender identities contribute to the marginalization and discrimination faced by MSM and transgender women [7,8]. These social factors can limit access to comprehensive sexual health education, healthcare services, and resources, increasing the likelihood of engaging in high-risk sexual behaviors and experiencing anal STI transmission.

In Brazil, TGW and MSM continue to face a disproportionately high burden of HIV infection. According to a study published in 2020, the prevalence of HIV among TGW was estimated to be around 31.6%, highlighting a severe vulnerability within this population. MSM also experience a significantly elevated risk, with an estimated HIV prevalence of 18.4%. These numbers underscore the urgent need for targeted HIV prevention and healthcare interventions to address the specific needs and challenges faced by TGW and MSM communities in Brazil, in line with international efforts to combat the HIV/AIDS epidemic.

There is scarce information on anal STIs among these vulnerable populations in Brazil, although the existing evidence suggests they present with a high prevalence of STIs in general. We aimed to describe the prevalence and associated factors of anal STIs in high-risk TGW and MSM populations attending an anal cancer preventive service for high-resolution anoscopy in Salvador, Brazil.

## 2. Methods

*Patients and setting:* this study evaluated the prevalence of STIs (*C. trachomatis—CT*, human papillomavirus—HPV, *N. gonorrhea—NG*, HIV-1, and syphilis) among MSM and TGW older than 18 years attending a referral public health service for the prevention of anal cancer through high-resolution anoscopy, at Federal University of Bahia Hospital in Salvador, Brazil, from February 2021 to June 2022. All participants were invited to fill in a questionnaire on their sexual behavior, sociodemographic information, and clinical symptoms. A blood sample and an anal swab were collected for STI testing.

*Laboratory tests:* molecular detection for HPV, NG, and CT was performed using GeneXpert kits (Cepheid, Solna, Sweden) on anal swabs from all participants. HIV screening was carried out using chemiluminescence and syphilis was assessed using VDRL, with any titration equal or higher than 1:8 considered positive. We used a 1:8 cut off because all but two VDRL-positive patients had a previous history of syphilis, and a positive treponemic test. All participants with a VDRL below that cut off had a previous history of syphilis treatment. The XpertHPV can detect the following HPV serotypes: HPV16, HPV18/45, and other high-risk HPV (31, 33, 35, 52, 58; 51, 59; 39, 56, 66, 68).

*Statistical analysis:* statistical analysis was performed using SPSS version 18.0 software [SPSS, IBM]. Categorical variables were expressed as absolute and relative frequencies and associations between the main variables were evaluated using the chi-square test or Fisher exact test, when appropriate.

## 3. Results

*Participants’ characteristics:* we included 141 participants in the study; 117 (82.9%) of them self-identified as MSM, 11 (7.8%) as bisexual men (BSM), and 15 (10.6%) as TGW. The majority (111/141, 78.7%) were older than 30 years, 89 (63.1%) had more than 12 years of schooling, 130 (92.2%) were single/divorced, 100 (70.9%) were currently employed, and 124 (87.9%) had a family income of up to five minimum wages. Overall, the mean age was 39.2 ± 10.4 years. MSM were older (39.9 ± 10.3 years) than BSM (37.9 ± 10.3 years) or TGW (34.9 ± 12.6 years), but the difference was not statistically significant (*p* = 0.2). Participants with a positive test for HPV were significantly older (37.8 ± 9.6 years) than negative ones (42.9 ± 11.9, *p* = 0.09). Age also reached a marginal significance in the comparison between participants declaring recent (last year) group sex (35.9 ± 7.6) and those who did not so (40.1 ± 10.9, *p* = 0.05).

*Prevalence of HIV and anal STIs:* among this study’s participants, 102 (72.3%) had a previous diagnosis of HIV, and 1 tested positive at the initial visit. Among people living with HIV (PLHIV), 89/117 (76.1%) self-declared to be MSM, 7/9 (88.9%) were BSM, and 5/15 (33.3%) were TGW. Anal infection by HPV (72.3%) was the most frequent diagnosis during the initial evaluation, followed by a positive syphilis serological test (22.6%) and CT detection (9.2%). All PLHIV were receiving treatment, but three of them had an HIV-1 RNA plasma viral load > 200 copies/mL (two had a viral load higher than 50,000 copies/mL and the third had 458 copies/mL).

At least one anal infection was detected in 112 (79.4%) participants. HPV was the most prevalent infection among TGW (86.7%), MSM (71.8%), and BSM (55,6%). Syphilis was more frequently detected among BSM (44,4%) and MSM (21.4%), while 20% of TGW had a positive VDRL. TGW had the highest prevalence for chlamydial infection (20.0%), but the lowest prevalence for syphilis (20.0%). There was a strong association between lower income (less than five minimum wages) and active anal STIs (*p* = 0.004, Fisher exact test). Table 1 summarizes the prevalence of anal STIs according to sociodemographic characteristics and HIV status of the study participants.

Participants with a positive HIV test had a significantly higher prevalence of syphilis than those testing negative (*p* = 0.002, Fisher exact test), but detection rates were similar for the other STIs, regardless of HIV serostatus. Table 2 displays the frequency of anal STIs according to the behavioral characteristics of study’s participants. The presence of at least one STI was also significantly associated with having had group sex in the last year (*p* = 0.04), the use of sexualized drugs (*p* = 0.02), or having had a higher number of sexual partners in the last 12 months (*p* = 0.04). Table 3 summarizes the main characteristics of the subjects with at least one STI detected during the evaluation.

## 4. Discussion

In the present work, we detected a high prevalence (79.4%) of at least one STI among MSM, BSM, and TGW. TGW presented the highest rates of HPV, gonorrhea, and chlamydia infections, but the lowest frequency of HIV. Of note, the low frequency of HIV among BSM and TGW may be biased by the low number of these attendees, 11 and 15, respectively. The main risk for active STIs were lower familial income, use of drugs during sex, and group sex/a higher number of sexual partners in the previous 12 months. In addition, PLHIV had a significantly higher prevalence of syphilis compared with HIV negative people.

Among the 102 participants who had a diagnosis of HIV infection, all but one knew his serostatus at initial evaluation. The fact that 80.8% of subjects with HIV infection presented with at least one STI is clear evidence that they engaged in unprotected sexual intercourse. The frequency of STIs (75.7%) among those who tested negative for HIV was similar of those who tested positive, and taken together, these findings demonstrate a high vulnerability to STIs among such populations, and the urgent need for strategies to improve preventive behavioral interventions. All PLHIV were on ART, which may give them the confidence to engage in sexual activity without the fear of HIV infection, as the U = U (undetectable = untransmissible) concept, which means patients on antiretroviral therapy presenting with HIV RNA undetectable plasma viral load do not transmit the infection during an unprotected sexual relationship, is well known to individuals on the treatment. This could explain the higher rate of syphilis diagnosis among this specific group.

The relationship between lower education and a higher risk of STIs was previously detected in a Brazilian study on PrEP early loss to follow up, suggesting a higher vulnerability of MSM and TGW to STIs, including HIV [9,10]. Another report, from the same group, showed that sexualized drug use was significantly associated with a higher risk of STIs and condomless anal sex, especially among TGW [11]. The main conclusions of this study indicate that sexualized drug use impacts vulnerability to STIs among TGW and young sexual and gender minorities. A higher average number of sexual partners, lower family income, and social discrimination were also associated with syphilis and HIV infections in MSM living in Salvador in a previous report, confirming the findings of the present work [12]. Authors also detected early sexual initiation as a predictive factor of higher vulnerability to HIV and syphilis, but we did not detect such an association in our study. These findings are also reported by different Brazilian studies, confirming that the detected risks factors are commonly found in several settings [13,14].

We detected at least one STI in all TGW, demonstrating the high vulnerability of this population. Laboratory-confirmed rates of STIs in the TGW population are scarce and can dramatically vary according to location and subpopulation [15,16].

Our study has some limitations like the small sample size and the inclusion of a population attending a single referral center for the prevention of anal cancer. However, our findings confirm that PLHIV engage in unprotected sexual activities despite their previous diagnosis of HIV infection, which is demonstrated by the high rate of syphilis in this specific group. In addition, the high frequency of STIs diagnosed in TGW also confirms the extreme vulnerability of this population to STIs, and the need for interventions to minimize such risk. As this was a cross-sectional study, we were not able to discriminate the time of HPV infection. However, even if the findings were associated with a persistent infection, indicating that these populations are at a high risk of acquiring HPV infection and developing anal cancer.

Efforts to address the prevalence and risk factors of anal STIs in MSM and transgender women require a multi-faceted approach. Comprehensive sexual health education programs that are inclusive, non-judgmental, and tailored to the specific needs of these populations are crucial [8,17]. Accessible and culturally competent healthcare services, including routine STI testing and treatment, can help to reduce the burden of anal STIs. Advocacy for LGBTQIA+ rights, challenging stigma and discrimination, and creating supportive environments are also essential components of addressing the underlying social determinants that contribute to the increased vulnerability of MSM and transgender women to anal STIs [18]. Most of the available evidence of STI in MSM and TGW comes from studies on PrEP, or are focused on specific infections. The present work demonstrates that even among subjects with a previous diagnosis of HIV, the prevalence of anal STIs is high, and clearly indicates a high vulnerability of MSM and TGW to acquiring such infections.

In conclusion, understanding the prevalence and risk factors associated with anal STIs in MSM and TGW is vital for effective prevention and management strategies. The elevated burden of anal STIs in these populations is influenced by individual-level behaviors, biological factors, and broader social determinants. Addressing these challenges requires comprehensive approaches that encompass sexual health education, accessible healthcare services, and efforts to combat discrimination and stigma. By promoting inclusivity, raising awareness, and providing necessary resources, we can work towards reducing the prevalence of anal STIs and improving the overall sexual health and well-being of MSM and transgender women.

## Figures and Tables

**Table 1:** Sociodemographic characteristics of 141 male individuals according to STI diagnosis at the clinic for anogenital diseases of HUPES, Salvador-Bahia, Brazil, 2021–2022.

Characteristics	Total (n =141)	Anal *C. trachomatis*	Anal *N. gonorrhea*	Anal HPV	Syphilis	At Least One STI
**Age (years)**	n (%)	n (%)	n (%)	n (%)	n (%)	n (%)
Mean (years ± SD)	39.2 ± 10.4	40.4 ± 9.3	37.2 ± 8.9	37.8 ± 9.6	40.3 ± 9.2	39.2 ± 10.5
≤30	30 (21.3)	2 (6.7)	1 (3.3)	23 (76.7)	6 (20.0)	25 (83.3)
>30	111 (78.7)	11 (9.9)	4 (3.6)	79 (71.2)	26 (23.4)	87 (78.4)
**Schooling**						
Less than 12 years	52 (36.9)	5 (9.6)	1 (1.9)	38 (73.1)	16 (30.8)	43 (82.7)
12 years or longer	89 (63.1)	8 (9.0)	4 (4.5)	64 (71.9)	16 (18.0)	69 (77.5)
**Marital status**						
Single/divorced	130 (92.2)	12 (9.2)	5 (3.8)	95 (73.1)	31 (23.8)	104 (80.0)
Married/stable union	11 (7.8)	1 (9.1)	0	7 (63.6)	1 (9.1)	8 (72.7)
**Currently employed**	100 (70.9)	10 (10.0)	4 (4.0)	75 (75.0)	22 (22.0)	81 (81.0)
Mean family income *						
Up to 5	124 (87.9)	12 (9.7)	4 (3.2)	90 (72.6)	29 (23.4)	100 (80.6)
>5	17 (12.1)	1 (5.9)	1 (5.9)	12 (70.6)	3 (17.6)	12 (70.6)
**Sexual orientation**						
MSM	117 (82.9)	9 (7.7)	4 (3.4)	84 (71.8)	25 (21.4)	92 (78.6)
Bisexual	9 (6.4)	1 (11.1)	0	5 (55.6)	4 (44.4)	7 (77.8)
TGW	15 (10.6)	3 (20.0)	1 (6.7)	13 (86.7)	3 (20.0)	13 (86.7)

* In 2022’s Minimum Wage.

**Table 2:** Distribution of anal sexually transmitted infections or positive serology for syphilis in an MSM and TGW population in Salvador, Brazil, according to HIV status.

	HIV		*p* Value
	Positive (n = 102)	Negative (n = 39)	
	n (%)	n (%)	
***C. trachomatis***	11 (10.7)	2 (5.3)	0.514 *
***N. gonorrhea***	4 (3.9)	1 (2.6)	1.0 *
**HPV**	75 (72.8)	27 (71.1)	1.0 **
Syphilis ***	30 (29.1)	2 (5.3)	0.003 *
**At least one STI**	84 (81.6)	28 (73.7)	0.429

* Fisher exact test; ** Chi-square test; *** VDRL ≥ 1/8.

**Table 3:** Association between the presence of at least one active STI and sexual behavior and practices in an MSM and TGW population in Salvador, Brazil.

Sexual Behavior and Practices	At Least One STI (n = 112) n (%)	No detected STI (n = 29) n (%)	*p* Value
**Sex for reward**	17 (15.2)	4 (13.8)	0.852 **
Group sex in the last year *	27 (24.1)	2 (6.9)	0.042 **
**Alcohol use before sex**	54 (48.2)	14 (48.3)	0.995 *
**Drug use before sex**	26 (23.2)	1 (3.4)	0.016 **
**Age at first sexual intercourse (n = 141)**			
Up to 14 years	38 (33.9)	11 (37.9)	0.687 *
>14 years	74 (66.1)	18 (62.1)	
**Number of male sexual partners in the last 12 months (n = 124)**			
Up to 10	79 (79.0)	23 (95.8)	0.040 **
>10	21 (21.0)	1 (4.2)	

* Chi-square test; ** Fisher exact test.

## Data Availability

Data will be available under request at: https://fbai.org.br/projetos/ (accessed on 23 October 2023).
